# LOW SENSORY RESPONSIVENESS IS ASSOCIATED WITH ACCELERATED AGING IN MID LIFE

**DOI:** 10.1093/geroni/igad104.0914

**Published:** 2023-12-21

**Authors:** Merav Asher, Maayan Agmon

**Affiliations:** University of Haifa, Haifa, HaZafon, Israel; University of Haifa, Haifa, HaZafon, Israel

## Abstract

The rate of aging is affected by genetic, behavioral, and emotional mechanisms; one possibly additional mechanism is the ability of the nervous system to sense and react appropriately to stimuli. This ability, called sensory responsiveness, may be a factor in shaping the aging mechanism and could be used as a marker of aging. The aim of this study was to examine the association between sensory responsiveness levels and rate of aging in midlife. A total of 97 participants born between 1974-1976 (age 45.63±0.67 at end point, 46% women) were included. Biological age was estimated at age ~32 and ~45 using the Klemera-doubal method and a set of biomarkers. Rate of aging was calculated by the difference in estimated biological age scores divided by passing years, reflecting temporal change in biological age. Sensory responsiveness was evaluated using the Sensory Responsiveness Questionnaire Scale, identifying high or low sensory responsiveness subtypes. We found that low sensory responsiveness was positively associated with rate of aging while controlling for chronological age, social engagement, and physical performance (linear regression, r=0.211; p=0.038). High sensory responsiveness subtype was not significantly associated with rate of aging (linear regression, r=-0.02; p=0.843). To conclude, low sensory responsiveness subtype may have a significant role in identifying the rate of aging in midlife. These findings highlight the importance of sensory responsiveness evaluations as a marker for rate of aging and the need for interventions focusing on sensory responsivity difficulties, as a mean of supporting healthier aging.

